# Mesenchymal stromal cells: promising treatment for liver cirrhosis

**DOI:** 10.1186/s13287-022-03001-z

**Published:** 2022-07-15

**Authors:** Lichao Yao, Xue Hu, Kai Dai, Mengqin Yuan, Pingji Liu, Qiuling Zhang, Yingan Jiang

**Affiliations:** Department of Infectious Diseases, Renmin Hospital of Wuhan University, Wuhan, 430060 People’s Republic of China

**Keywords:** Liver cirrhosis, Mesenchymal stromal cells, Trans-differentiation, Paracrine effects, Immunomodulatory effects, Exosome

## Abstract

Liver fibrosis is a wound-healing process that occurs in response to severe injuries and is hallmarked by the excessive accumulation of extracellular matrix or scar tissues within the liver. Liver fibrosis can be either acute or chronic and is induced by a variety of hepatotoxic causes, including lipid deposition, drugs, viruses, and autoimmune reactions. In advanced fibrosis, liver cirrhosis develops, a condition for which there is no successful therapy other than liver transplantation. Although liver transplantation is still a viable option, numerous limitations limit its application, including a lack of donor organs, immune rejection, and postoperative complications. As a result, there is an immediate need for a different kind of therapeutic approach. Recent research has shown that the administration of mesenchymal stromal cells (MSCs) is an attractive treatment modality for repairing liver injury and enhancing liver regeneration. This is accomplished through the cell migration into liver sites, immunoregulation, hepatogenic differentiation, as well as paracrine mechanisms. MSCs can also release a huge variety of molecules into the extracellular environment. These molecules, which include extracellular vesicles, lipids, free nucleic acids, and soluble proteins, exert crucial roles in repairing damaged tissue. In this review, we summarize the characteristics of MSCs, representative clinical study data, and the potential mechanisms of MSCs-based strategies for attenuating liver cirrhosis. Additionally, we examine the processes that are involved in the MSCs-dependent modulation of the immune milieu in liver cirrhosis. As a result, our findings lend credence to the concept of developing a cell therapy treatment for liver cirrhosis that is premised on MSCs. MSCs can be used as a candidate therapeutic agent to lengthen the survival duration of patients with liver cirrhosis or possibly reverse the condition in the near future.

## Background

Liver disease is an infection of the liver induced by viruses (such as hepatitis B and C), autoimmune hepatitis, alcoholic steatohepatitis (ASH), non-alcoholic steatohepatitis (NASH), and progressive metabolic diseases resulting in liver failure, cirrhosis, and liver cancer. It has become an increasingly serious cause of death worldwide, accounting for 3.5% of all annual mortality globally [1]. Long-term liver injury gradually results in the loss of liver function and accumulation of extracellular matrix (ECM), leading to the occurrence of liver fibrosis. The end stage of liver fibrosis is cirrhosis, and patients with decompensated cirrhosis develop multiple complications; the most common clinical manifestations are ascites and gastroesophageal variceal bleeding. Late complications include jaundice, coagulopathy, hepatic encephalopathy, acute kidney injury, and hepatorenal syndrome (HRS), with complications recurring with increasing frequency after the initial presentation, and most patients die within a median time of approximately 2 years [2]. The only available option is liver transplantation, but its clinical use is restricted by donor scarcity and immune rejection. Therefore, there is an urgent need for effective treatment strategies to reverse cirrhosis.

Recently, mesenchymal stromal cells (MSCs) have received much attention in many different areas of health and medical research. MSCs are thought to be potentially useful and appropriate candidates for treating acute liver failure and cirrhosis owing to their ability to differentiate into hepatocyte-like cells (HLCs) and immunomodulatory properties [3–6]. Clinical experiments have shown that treatments based on MSCs are both safe and feasible to use for a wide variety of disorders, including autoimmune diseases, cancer, Crohn’s disease, respiratory disorders, liver cirrhosis, multiple sclerosis, spinal cord injury, diabetes and its complications, bone and cartilage injuries, osteoarthritis, heart diseases, and graft-vs-host disease [7]. MSCs are defined as multipotent stromal cells with self-renewal capacity that could be readily extracted from a wide range of tissues (e.g., amniotic fluid, umbilical cord, adipose tissue, bone marrow, and menstrual blood) and amplified in vitro [8]. MSCs do not express major histocompatibility complex (MHC) antigens of the class II subtype and contain low levels of MHC molecules of the class I subtype. MSCs also lack the co-stimulatory molecules essential for immune detection, including CD40, CD80, and CD86. Therefore, MSCs generally have low immunogenicity and can avoid immune rejection by the recipient, which serves as the foundation for their allogenic application [9]. The therapeutic advantages of MSCs include self-renewal, homing to the site of injury, immunomodulation, multidirectional differentiation, and secretion of trophic factors that promote repair and regeneration of damaged tissues, and the use of MSCs is free of any ethical concerns [10]. Since the first isolation of MSCs from the bone marrow of a mouse in 1976, several fundamental and clinical research trials have revealed that MSCs may enhance liver function and treat liver cirrhosis in a way that is both safe and effective [9]. As of Dec. 2021, there have been a total of 60 registered clinical studies involving MSCs in liver illness treatment, with 45 of those trials focusing specifically on liver cirrhosis(www.clinicaltrials.gov) (Table1).

**Table 1:** Completed clinical trials using MSCs transplantation to treat liver cirrhosis, registered under ClinicalTrials.gov

NCT number	Dates	Conditions	Study phase	Interventions	Cell source	No. of patients	Primary outcome measures	Locations
NCT01342250	2010.10–2011.10	Liver Cirrhosis	Phase 1 Phase 2	Conventional therapy plus hUC-MSCs treatment	hUC-MSCs	20	Overall Survival	China
NCT01591200	2021.06–2016.04	Alcoholic Liver Cirrhosis	Phase 2	Allogeneic MSCs injected through the hepatic artery	BM-MSCs	40	Safety	India
NCT01875081	2012.11–2016.03	Alcoholic Liver Cirrhosis	Phase 2	5*10^7^ autologous BM-MSCs injected through the hepatic artery	BM-MSCs	72	Histopathological evaluation	Korea
NCT01220492	2009.05–2016.04	Liver Cirrhosis	Phase 1 Phase 2	Taken i.v. once per 4 weeks, at a dose of 0.5*10^6^ MSC/kg body for 8 weeks	UC-MSCs	266	1.Survival time; 2. Incidence of HCC events	China
NCT00420134	2006.02–2009.06	Liver Failure Cirrhosis	Phase 1 Phase 2	Progenitor of hepatocyte derived from Mesenchymal stem cell injected into portal vein	From the end-stage liver disease	30	1.Liver function test; 2. MELD score	Iran
NCT04243681	2019.07–2020.09	Liver Cirrhosis	Phase 4	MSCs and Hematopoietic Stem cell injected through hepatic artery	CD34 + cell and MSCs	5	Safety	India
NCT01454336	2010.06–2013.07	Liver Fibrosis	Phase 1	Autologous MSCs injected via portal vein; 30 mg pioglitazone daily for 24 months	BM-MSCs	3	1. ALT, AST, Serum Albumin levels; 2. The decrease in grade of liver fibrosis	Iran
NCT01062750	2012.10–2015.05	Liver Cirrhosis	Not Applicable	Autologous AT-MSCs via intrahepatic arterial catheterization	AT-MSCs	4	All cause harmful events	Japan

Patients with liver cirrhosis may experience an improvement in their liver function following MSCs treatment, which appears to be safe and have a good safety profile and is generally well tolerated. Results obtained in a four-case clinical report illustrated that transplantation of autologous bone marrow mesenchymal stem cells (BM-MSCs) via peripheral vein is safe and the Model for End-Stage Liver Disease (MELD) scores in two patients with decompensated liver cirrhosis is improved [11]. The transplantation of umbilical cord mesenchymal stem cells, often abbreviated as UC-MSCs, was evaluated in a phase I–II clinical study encompassing patients who had hepatitis B cirrhosis. The intravenous infusion of UC-MSCs resulted in a considerable reduction in the volume of ascites, improvements in liver function (such as serum bilirubin and serum albumin levels), and a reduction in the MELD Na score, without any serious adverse reactions or complications [12]. In phase I–II clinical trial by Pedram et al. [13], autologous MSCs were amplified in vitro and differentiated into hepatocytes and then injected via the portal or peripheral vein into patients with the end-stage liver disease having the MELD score ≥ 10. Liver function improved after MSCs transplantation as evidenced by the lowered MELD score, and all of the patients tolerated the therapy. Peng et al. [14] indicated that patients who suffered from liver failure due to hepatitis B and underwent autologous bone marrow mesenchymal stem cell transplantation demonstrated satisfactory short-term effectiveness (from the fourth week to the thirty-sixth postoperatively), but did not significantly enhance the long-term prognosis of these individuals. Despite this, other clinical investigations demonstrate that there is no enhancement in liver function following the transplantation of MSCs [15, 16]. There is a need for more extensive trials to establish that the transplantation of MSCs into patients with liver cirrhosis is both safe and effective.

## Pathogenesis of liver cirrhosis

Liver cirrhosis refers to the advanced stage of liver fibrosis, which may be induced by several diseases and disorders that affect the liver, such as chronic alcoholism and hepatitis. It is the consequence of an excessive accumulation of extracellular matrix (ECM) and collagen I in response to chronic damage [17]. The process via which liver fibrosis develops might vary considerably depending on the causes, which include hepatitis virus, alcohol, or bile acids. In most cases, the initial stage includes damage to liver cells, which results in the generation of oxygen-free radicals and inflammatory substances. Subsequently, Kupffer cells and other inflammatory cytokines become activated and are recruited into the process. The next step is the activation of hepatic stellate cells (HSCs) [18]. This is the general process underlying liver fibrosis. HSCs, which are found in the space of Disse, perform an integral function in the onset and progression of liver fibrosis.

**Fig. 1:**
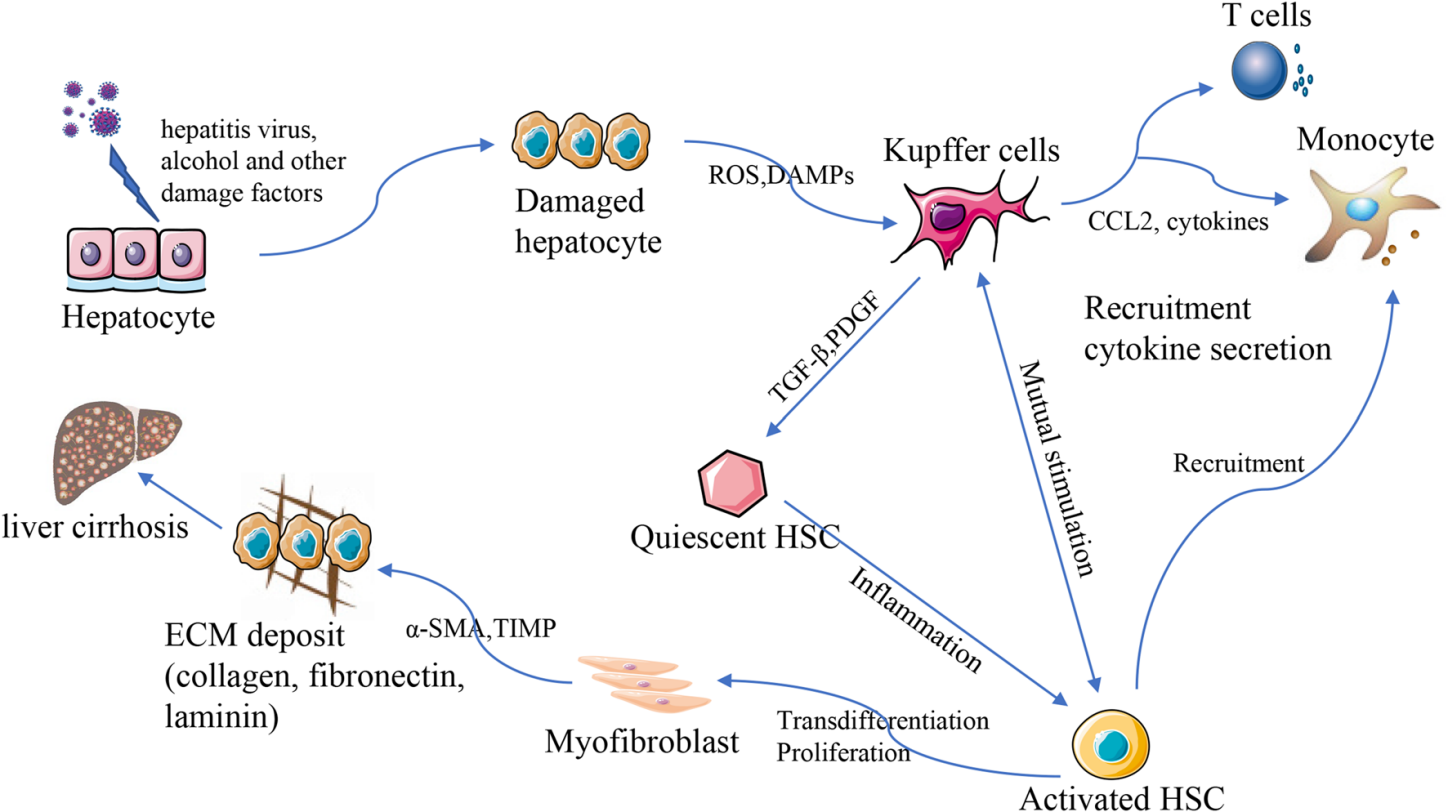
Pathogenesis of liver cirrhosis. Liver fibrosis is initiated by hepatic injury and the subsequent imbalance of ECM synthesis and degradation mediated by activated HSCs. Cirrhosis is the most advanced stage of liver fibrosis. ECM, extracellular matrix; HSCs, hepatic stellate cells; TIMP, tissue inhibitors of metalloproteinase; PDGF, platelet-derived growth factor; TGF-β, transforming growth factor-β

During chronic liver injury, Kupffer cells release various inflammatory cytokines (e.g., platelet-derived growth factor (PDGF), transforming growth factor-β (TGF-β)) that activate HSCs, causing them to trans-differentiate into myofibroblasts [19]. Kupffer cells also stimulate the influx of bone marrow-derived immune cells into the liver by releasing CCL2 and CCL5, where they develop into Ly-6C + macrophages that promote inflammation, angiogenesis, and fibrogenesis, driving the progression of fibrosis [20]. Inflammatory cytokines induce the trans-differentiation of HSCs from a quiescent to a proliferative, migratory, and fibrotic phenotype (myofibroblast). This leads to the generation of plenty of extracellular matrix (ECM) as well as the expression of α-smooth muscle actin (α-SMA) [21]. TGF-β is the main cytokine in the stimulation of HSCs trans-differentiation and the signal of epithelial-to-mesenchymal transitions (EMT). The TGF-β1 activation enhances ECM synthesis and suppresses ECM degradation, thereby speeding up the advancement of liver fibrosis [22]. Myofibroblasts are known to produce tissue inhibitors of matrix metalloproteinases (TIMP) to prevent the degradation of ECM by matrix metalloproteinases (MMP) and maintain the integrity of ECM [23] (Fig. 1).

## Characteristics of MSCs

MSCs are multipotent fibroblast-like cells that are often extracted from the umbilical cord, dental pulp, adipose tissue, menstrual blood, and bone marrow [24, 25]. The International Society for Cell therapy (ISCT) established a set of minimum criteria to characterize MSCs in the year 2006: (1) cells must be plastic-adherent; (2) CD90, CD73, and CD105 must be expressed in the MSCs population, whereas HLA class II, CD79a, or CD19, and CD14, CD34, CD45, or CD11b must not be expressed; and (3) it is necessary for the cells to have the ability to differentiate into osteoblasts, chondrocytes, and adipocytes [26]. Owing to their flexibility, several studies suggest that MSCs may also develop into cells of endodermal (hepatocytes) or neuro-ectodermal (oligodendrocytes, astrocytes, neurons) origin [27]. Besides their ability to differentiate, MSCs possess at least two other properties that contribute to their beneficial therapeutic value in the treatment of immunological-mediated disorders: homing to the site where tissues are damaged and modulation of the immune response [28]. Inflammatory cytokines including interferon gamma (IFN-γ), interleukin (IL)-1, and tumor necrosis factor alpha (TNF-α) produced following tissue injury and during inflammation stimulate cell surface expression of adhesion molecules that facilitate rolling and migration of MSCs into ECM.

It would seem that the soluble products of MSCs, which include extracellular vesicles (EVs), cytokines, trophic factors, and chemokines, are responsible for their major therapeutic impacts, which include angiogenic, antioxidant, anti-fibrotic, and anti-inflammatory properties. MSCs also regulate innate and adaptive immune responses through intercellular contact (binding of programmed death 1 (PD-1) to its ligands PD-L1 and PD-L2) or paracrine mechanisms [29]. For example, hepatocyte growth factor (HGF) and IL-6 produced by MSCs impede the differentiation of monocytes into dendritic cells, lowering their propensity to cause inflammation, decreasing the secretion of the pro-inflammatory cytokines IL-12 and IFN-γ, and increasing the production of anti-inflammatory cytokines IL-10, sequentially weakening the activation of T cells [30]. MSCs inhibit the Kupffer cell activity, which leads to an attenuation in the production of the pro-inflammatory cytokine TNF. In addition, MSCs transform pro-inflammatory M1 macrophages into anti-inflammatory M2 macrophages by secreting prostaglandin E2 (PGE2) [31]. Besides, MSCs can modulate the immunological response by triggering the Notch 1 signaling pathway, generating HLA-G5, PGE2, and TGF-β1 and promoting the activation and expansion of CD4 + CD25 + FoxP3 + regulatory T cells (Tregs). By producing indoleamine 2,3-dioxygenase (IDO) and heme oxygenase 1, MSCs suppress the proliferative ability of CD8 + T lymphocytes and enhance the conversion rate of CD4 + T lymphocytes from T-helper 1 to T-helper 2 phenotypes [32].

The decrease of inflammation is by far the most common use of MSCs [33]. However, MSCs are not inherently immune suppressants; rather, they need a “licensing” step to be undertaken by the acute phase inflammatory molecules, such as TNF-α and IFN-γ, or toll-like receptor (TLR) ligands [34]. Recent research has shown that mesenchymal stem cells (MSCs) only become immunosuppressive when they are exposed to sufficiently high levels of pro-inflammatory cytokines [29, 35, 36]. MSCs have the potential to exhibit a pro-inflammatory phenotype when they are exposed to low levels of IFN-γ and TNF-α. They do this by producing chemokines (such as CXCL9 and CXCL10), which bring lymphocytes to the regions of inflammation, thereby enhancing the immune response of T cells [29]. In contrast, when MSCs are stimulated with high levels of IFN-γ and TNF-α, there is an improvement in the production of inhibitory soluble substances, and MSCs acquire an anti-inflammatory phenotype and inhibit the activation as well as effector properties of inflammatory dendritic cells (DCs), T lymphocytes, macrophages, natural killer (NK) cells, and NKT cells [37]. This suggests that MSCs suppress or promote inflammation depending on the pathological conditions to which they are exposed and that the levels and concentrations of inflammatory cytokines are fundamental factors influencing the immunomodulatory capacity of MSCs [38, 39]. Li et al. [29] confirmed that MSCs may stimulate immunological responses when they are exposed to a low level of pro-inflammatory cytokines or when iNOS activity is absent. It has been suggested that iNOS, which is present in mouse cells, and IDO, which is present in human cells, might act as a molecular switch between the immunosuppressive and immune-enhancing properties of MSCs [40]. TLRs priming can critically impact the multilineage potential, phenotype, and immunomodulation ability of MSCs [41]. The transition from a pro-inflammatory to an anti-inflammatory phenotype could also be dependent on the degree to which MSCs are stimulated by the TLRs that are expressed on their surface. Activation of TLR4 that is dependent on lipopolysaccharide may affect the polarization toward a pro-inflammatory phenotype, which is crucial for early damage responses, while the stimulation of TLR3 that is based on double-stranded RNA (dsRNA) might trigger a polarization toward an anti-inflammatory type [42]. The maintenance of a stable equilibrium between these antagonistic pathways might, on the one hand, help to stimulate the host's immune response, while, on the other hand, it may generate a feedback loop that inhibits extensive tissue damage and stimulates regeneration.

It has been demonstrated that MSCs, like immune cells, can "remember" a stimulation even after being exposed to other settings [40]. IIFN-γ activates the transcription and synthesis of IDO, HGF, and TGF-β in MSCs through JAK/STAT pathway [43]. According to Saldana and her colleagues' study [44], the pro-inflammatory cytokine TNF-α acts as a priming factor for MSCs, and priming MSCs using a medium that had been conditioned by either pro- or anti-inflammatory macrophages enhanced their immunoregulatory capacity by increasing the amount of PGE2 that they secreted. As a result, MSCs have been primed to induce a “short-term memory” effect in vitro by imitating the stimuli that are present in the microenvironment; therefore, it is not necessary to activate the MSCs in vivo to achieve the specific therapeutic activities that are being sought [45].

## Mechanisms of MSCs-based therapy in liver cirrhosis

**Fig. 2:**
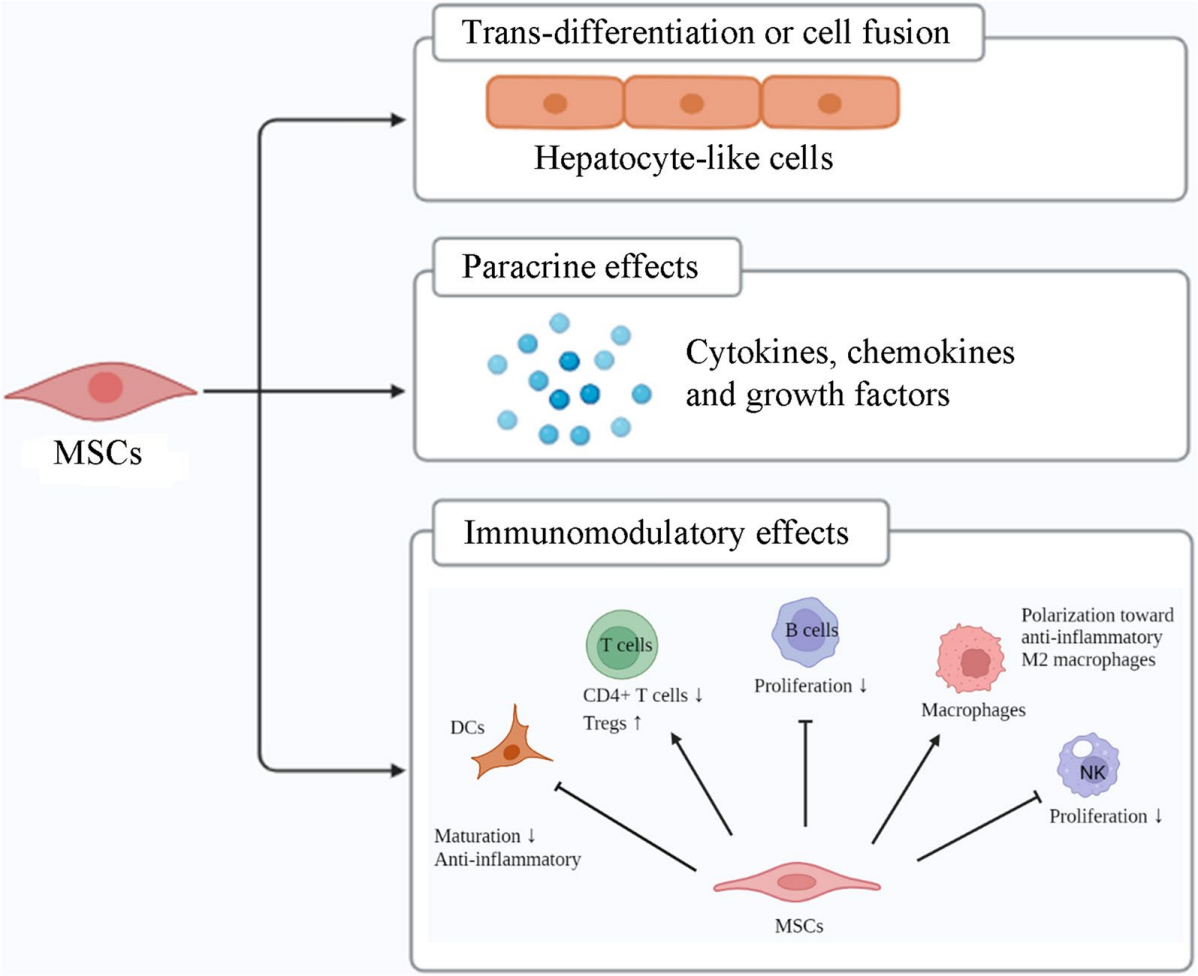
The potential mechanisms of MSCs in liver cirrhosis

The mechanisms of MSCs in the treatment of cirrhosis have been investigated from multiple perspectives in basic research and are broadly divided into three types: (1) When introduced into damaged liver tissues, MSCs have the potential to either differentiate into hepatocytes or fuse with existing hepatocytes, making them a useful resource for the regeneration and repair of hepatic tissues; (2) MSCs are capable of producing a wide range of cytokines and growth factors, and they may also have the ability to exert a paracrine impact, which helps to stimulate the repopulation of endogenous cells in injured tissues; and (3) MSCs have a suppressive influence on many other types of cells, such as natural killer cells (NKs), B lymphocytes, and T lymphocytes which allow them to exert an immunoregulatory impact on liver illnesses [46, 47] (Fig. 2).

## Trans-differentiation versus cell fusion

The majority of investigations using HLCs, generated from MSCs, have reported encouraging findings indicating that these cells both improved their serum parameters and recovered the liver function in vivo [48]. To this day, the trans-differentiation of MSCs into HLCs has mostly been induced by the use of four primary methods, which include the addition of cytokines as well as growth factors, alterations to physical parameters, modification of the microenvironment, and genetic alteration [49]. In 1996, Abe et al. [50] revealed that mouse ESCs could differentiate into endodermal cells. In a later study, Hamsaki et al. [51] discovered that certain growth factors may be used to direct mouse embryonic stem cells to differentiate into HLCs. The cytokine combination approach is one of the available ways of induction, and it has received a great deal of research. The origin of the MSCs has a significant impact on their ability to differentiate as well as the kinds of growth factors and cytokines needed to induce it [49]. There is no universally accepted standard for the growth factor cocktail; its composition will vary according to the origin of the MSCs as well as the characteristics of each study. Lee et al. [5] designed a protocol to differentiate human UC-MSCs and BM-MSCs into HLCs: MSCs were treated for 7 days using a differentiation medium (comprising nicotinamide, bFGF, and HGF), before treatment with a mature medium (comprising transferrin and selenium (ITS), dexamethasone, insulin, oncostatin M (OSM)) to induce differentiation. It is important to note that some researchers favor the use of sequential differentiation step by step. This involves the use of a variety of biochemicals, cytokines, and growth factors that each play a role in the many stages of development and regeneration. In most cases, a sequential administration of varying levels of EGF, OSM, and HGF, together with insulin and glucocorticoids, in a culture medium that is optimized for hepatocytes is employed [52]. FGF and EGF are the two growth factors that are responsible for inducing MSCs into endodermal cells during the primary induction stage [53]. OSM and dexamethasone are required to induce further maturation, together with the addition of FGF, ITS, and HGF [54].

After culturing hUC-MSCs for 16 days in a mixture containing sodium selenite, insulin, dexamethasone, bFGF, and HGF, Zhao et al. transplanted the cells into a medium supplemented with OSM. The hUC-MSCs that were generated as a consequence displayed a strong capacity for hepatic differentiation as well as activities that are particular to hepatocytes [55]. Raufi and colleagues conducted a study in which they differentiated umbilical cord vein MSCs into hepatic MSCs by employing OSM and HGF in a two-step strategy, followed by a 4-week induction. An immunological examination revealed that the generated HLCs produced protein biomarkers that are specific to the liver, and they also displayed certain properties that are typical of hepatocytes [56]. Si-Tayeb et al. [57] illustrated that human iPSCs may be differentiated into functional hepatocytes by following a 4-step differentiation strategy and maintaining a low oxygen level. This was accomplished via lentiviral transduction of the generated LN28, NANOG, SOX2, and OCT3/4. They revealed the proliferation of these cells in the fetal liver of mice for seven days following transplantation. Research by Ang et al. [58] indicated that iPSC-derived HLCs exhibited different hepatocyte activities in vitro at day 18 of differentiation, possessed CYP3A4 enzymatic activity, experienced positive staining for periodic acid Schiff in addition to the liver-specific surface marker ASGR1, and expressed hepatocyte markers, including albumin, AAT, and CPS1. Most importantly, in the FRG murine model of hereditary tyrosinemia, iPSC-derived HLCs transplantation resulted in an elevation in their short-term survival rate. Zhou et al. [48] proved that by transfecting MSCs with a combination of five miRNAs, they can be quickly and effectively transformed into functional HLCs. Additionally, intravenous transplantation of the HLCs was shown to produce urea, store glycogen, take up LDL, and enhance the status of CCl_4_-induced fulminant liver failure and acute liver injury mouse models. Mun et al. [59] developed a novel human iPSC-derived hepatocyte-like liver organoids that are anatomically identical to and with functional performance comparable to liver organoids derived from adult tissues while simultaneously preserving their mature hepatic features during long-term culture, thus generating a strong hepatic model in a way that is both reproducible and reliable for toxicity prediction, screening of drugs, regenerative and inflammatory responses, as well as modeling for disorders including hepatic steatosis. Collectively, numerous parameters, such as the timing, dosage, and particular type of growth factors, have been proven to alter hepatic induction. Furthermore, diverse sources of MSCs may be involved in the trans-differentiation process into HLCs. Unfortunately, the data that are now available cannot provide insights into which source or technique is superior for producing functioning HLCs to address the necessity for a viable source for transplantation.

Hepatocytes in hepatic chords reside in three-dimensional (3D) formations within the original liver matrix. These hepatocytes are linked to each other by tight and gap junctions, and they are located proximal to non-parenchymal cells [60]. When compared with traditional two-dimensional (2D) cultures, 3D arrangements, such as organoids or spheroids, have many advantages, including the capacity of stem cells to differentiate and the preservation of metabolic activity [61]. Some research teams created artificial 3D scaffolds intending to model the 3D milieu and morphology of the liver. These teams then studied whether or not the artificial scaffolds were able to promote MSCs differentiation more effectively as opposed to 2D culture. Gieseck and his colleagues developed a culture approach for the iPSC-derived HLCs maturation that makes use of 3D collagen matrices which were compatible with high-throughput screening. In comparison with traditional 2D structures, this culture approach considerably accelerates the functional development of HLCs into a mature adult phenotype [62]. Saito et al. [63] used human adipose-derived MSCs to generate both 2D- and 3D-cultured HLCs. They discovered that 3D-cultured HLCs were more comparable to primary hepatocytes and demonstrated significantly superior hepatocellular functions when contrasted to 2D-cultured HLCs. Since liver regeneration in vivo is linked to portal pressure, which characterizes fluid shear stress, shear stress, as well as the fluid friction force created by a continuously flowing fluid, can considerably alter the hepatic differentiation of MSCs [64]. Yen et al. [65] developed an innovative microfluidic system combined with the hepatic differentiation protocol, which may generate HLCs from MSCs in a more effective manner and with a more quick functionality maturation in comparison with a typical static culture method.

However, according to the findings of certain research, there is currently no differentiation method that can generate HLCs that are capable of exerting most hepatic activities at a level comparable to that of an adult liver. In the scientific literature, it is frequently noted that the HLCs phenotype is comparable to that of neonatal or fetal hepatocytes. When compared with primary hepatocytes, the level of expression of several genes that are favored by hepatocytes is much lower in HLCs [60, 66]. Orge et al. [67] found that the gene expression profiles of MSCs-derived HLCs were distinct in comparison with that of primary hepatocytes, even though these HLCs demonstrated certain levels of hepatocyte activity, showing a combination of characteristics associated with immature progenitors and mature hepatocytes. It is important to note that these cells were unable to mature in vivo in the Ah^cre^Mdm2^fl/fl^ murine model that was established for the regeneration of hepatocytes. After isolating UC-MSCs and differentiating them into HLCs, Campard et al. [68] made a comparison of the results of their study with undifferentiated UC-MSCs and also freshly extracted liver cells. They demonstrated that HLCs had developed some of the functional characteristics of hepatocytes, including the ability to store glycogen and generate urea, as well as active G6P and CYP3A4 enzymes. However, certain hepatic biomarkers including hepatocyte nuclear factor 4 (HNF-4) and hepPar1 could not be identified, demonstrating that the differentiation had not progressed to the point of mature hepatic cells. In the field of regenerative medicine, one of the most huge challenges right now is the synthesis of HLCs from stem cell sources. More efforts are needed to develop a protocol to differentiate MSCs into HLCs with the same function as adult mature hepatocytes.

There has been a great deal of discussion regarding the ways through which stem cells trans-differentiate into new mature hepatocytes. Based on the data from the recent experiments, it is plausible to conclude that trans-differentiation is a very uncommon and unphysiological process, taking place either over a long duration or just in the context of well-controlled experiments [69]. It has been shown in a growing body of research and reports that the transplantation of cells taken from bone marrow may lead to the production of hepatocytes that are fully functioning. The mechanism of this process has been identified as the cellular fusion between bone marrow-derived cells and host hepatocytes [70, 71]. Wang and his colleagues discovered that in the fumarylacetoacetate hydrolase (FAH)-deficient murine liver, bone marrow cells adopt hepatocyte morphologies through cell fusion [72]. A cytogenetic investigation of BM-derived hepatocytes extracted from host livers based on research on the transplantation of bone marrow from female Fah^+/+^ mice into lethally irradiated Fah^−/−^ males revealed an abundance of (80, XXXY) and (120, XXXXYY) hepatocyte karyotypes, corroborating the hypothesis that cell fusion is a frequent event in the process of producing hepatocytes that express FAH [73]. The restoration of hepatic functionality by the fusion of donor cells obtained from the bone marrow or another location with local hepatocytes offers exciting insights into the possible use of these cells in the development of future regenerative liver treatment therapies.

## Paracrine effects

Although interest in the early treatment of MSCs revolves around their ability to differentiate in the liver, there is a growing body of research that provides significant support for the assumption that the activities of MSCs are primarily mediated via paracrine processes instead of through trans-differentiation [74]. According to the findings of Parekkadan and his colleagues, differentiation of engrafted MSCs into hepatocytes occurs rarely, although the factors released by transplanted MSCs have a significant and favorable influence on hepatocytes. These researchers were able to effectively repair mice models of acute liver damage by using molecules that were derived from MSCs [75]. In order to avoid HSCs from being activated and causing liver fibrosis, MSCs release a vast variety of antiapoptotic growth factors, including VEGF, HGF, and IGF-1 [76]. Haldar and his colleagues encapsulated human BM-MSCs in an alginate–polyethylene glycol hybrid hydrogel that is permeable to soluble factors (cytokines, glucose, and oxygen), although it is impermeable to antibodies and does not allow for direct cellular interactions. They also observed that injecting microencapsulated MSCs into mouse models of chronic liver damage alleviated inflammation and liver fibrosis, which suggests that the benefits may be completely attributable to substances produced by MSCs [23]. In vitro experiments have demonstrated that the coculture of MSCs with HSCs inhibits the proliferation and activation of HSCs, reduces α-SMA expression by secreting IL-10 and TGF-β, and induces HSCs apoptosis via the release of nerve growth factor (NGF) and HGF [7, 77]. It has been found that the conditioned medium (CM) of MSCs considerably suppresses hepatocyte apoptosis and enhances hepatocyte proliferation in various mouse models of acute liver damage [78]. Meier et al. [79] found that MSCs-CM decreased the expression of MMP-2, α-SMA, and type I collagen from primary HSCs, indicating that the secretion factors of MSCs can prevent HSCs activation. Although researchers identified evidence supporting paracrine impacts following MSCs transplantation and MSCs synthesize a broad range of cytokines and growth factors, the particular mechanisms and the corresponding molecular pathways still need additional exploration.

## Immunomodulatory effects

The immunoregulatory properties of MSCs have been the focus of many research reports in both in vitro and in vivo settings. Several studies provide significant evidence that the therapeutic benefits of MSCs in chronic and acute liver disorders are systemic and that these impacts are dependent on the secretion of substances that are trophic and immunoregulatory [80]. MSCs exert their immunotherapy impact by regulating both innate and adaptive responses [81]. MSCs were able to inhibit the growth of T cells by producing soluble substances, notably, TGF-β, PGE2, IDO, NO, and HGF. Via the mechanism of the production of TGF-β, MSCs not only have the capacity, but also the propensity, to stimulate the differentiation of CD4^+^ T cells into CD25^+^Foxp3^+^ regulatory T cells (referred to as induced Tregs) [82]. According to the findings of Zhang et al. [83], MSCs greatly reduced the amount of CD4^+^ T cells infiltrating the liver, the proportion of CD4^+^ T lymphocytes that were activated, as well as the overall concentration of Th1 cells, which was followed by the induction of regulatory DCs and Tregs in the liver in order to ameliorate the damage caused to the liver. MSCs drastically alleviated CCl_4_-mediated liver fibrosis by lowering the proportion of Th17 cells and elevating the levels of CD4^+^IL-10^+^ T cells as well as the levels of immune suppressive factors, including kynurenine, IDO, and IL-10 [84]. In addition, MSCs enhanced liver functionality and ameliorated the clinical symptoms in patients with hepatitis B virus-mediated decompensated liver cirrhosis by remarkably downregulating the expression levels of IL-6 and TNF-α while simultaneously upregulating the expression level of IL-10 [85]. Successful immunoregulation, as well as tissue regeneration, depends on interactions between MSCs and macrophages, particularly paracrine modulation and direct cell interaction [86]. Inflammatory cytokines produced by M1 macrophages or activated T lymphocytes might stimulate MSCs and cause the production of cytokines that alter the monocyte differentiation toward an anti-inflammatory phenotype and, eventually, toward M2 macrophages [87]. MSCs have previously been shown to stimulate liver infiltration by host monocytes and neutrophils, leading to the alleviation of fibrosis through MMP synthesis [88]. Luo et al. [89] revealed that the transplantation of BM-MSCs stimulated M2 macrophages, which expressed MMP13, while simultaneously inhibiting M1 macrophages, which subsequently inhibited the activation of HSCs, eventually exhibiting a synergistic effect in reducing the severity of the liver fibrosis caused by CCl_4_. The invasion of immune cells is a necessary stage on the path to liver damage. MSCs provide an immunotolerant milieu in liver tissue by inhibiting the engagement of pro-inflammatory immune cells while enhancing the recruitment of anti-inflammatory immune cells, thereby eliminating acute or chronic liver damage [90].

MSCs are known to release a variety of anti-inflammatory substances in conjunction with a variety of pro-inflammatory cytokines such as IL1b, IL-6, IL-8, and IL-9, which mediate its immunomodulatory effect [91]. As we mentioned above that MSCs suppress or promote inflammation depending on the pathological conditions to which they are exposed. Waterman et al. [42] proved that the activation of T lymphocytes was enhanced when MSCs were transiently exposed to 10 ng/ml LPS for less than one hour in the coculture test. In comparison, MSCs that had been subjected to poly I: C at a concentration of 1 μg/ml were able to inhibit the activation of T lymphocytes while simultaneously boosting the expression of IDO and PGE2. The study by Lin et al. [36] indicated that a protective anti-inflammatory response was triggered in macrophages by MSCs subjected to LPS at concentrations ranging from 1 to 20 μg/ml. IFN-γ is primarily generated by activated T-helper 1 (Th1) cells and serves as an essential modulator of the innate as well as the adaptive immune responses. According to the findings of Ren and his colleagues, the presence of IFN-γ in combination with one of the pro-inflammatory cytokines (IL-1β, IL-1α, or TNF-α) is necessary for the inhibition of T-lymphocyte activation that is mediated by MSCs [92]. Thus, the ultimate immunomodulatory impact may be determined by the balance between anti-inflammatory and pro-inflammatory cytokines in the milieu in which MSCs reside.

In the past decades, MSCs have gained a lot of attention and investigation, both in the laboratory and in clinical settings owing to their numerous benefits. Although MSC-related clinical studies in patients diagnosed with liver cirrhosis are shown to be safe and effective in the short term, the favorable benefits of these trials have been shown to diminish over time or have not been assessed at all. According to the findings of Lotfinia and colleagues, MSC-CM can improve the histological and biochemical characteristics of livers, but it does not significantly improve the survival of murine with TAA-mediated liver failure [93]. In patients with decompensated cirrhosis, autologous BM-MSCs transplantation possibly does not exert a therapeutic impact, according to the findings of one randomized controlled trial [16]. Additionally, there are still a lot of challenges that need to be resolved, such as the ideal timing, optimum delivery channel, and adequate cell count for MSCs transplants. Therefore, additional research on the betterment of the engraftment and MSCs survival rate in the liver is required to enhance the effectiveness of MSCs treatment. These studies should also identify methods to enhance the long-term implantation of mesenchymal stem cells in the host liver. What is more, the clinical application of MSCs in the treatment of liver disease is just in its infant stages at this point, and large-scale randomized and controlled clinical trials need to be conducted with longer follow-up periods to enhance the dependability of the clinical safety and effectiveness of MSCs for human liver disorders.

## Pretreatments enhance the therapeutic effects of MSCs in liver cirrhosis

After being isolated and cultured in vitro, MSCs fail to maintain their capacity for subsequent applications because they are deprived of nutrients and oxygen [94]. External growth factors are also unable to preserve MSCs' capabilities. While MSCs are capable of differentiating into a wide variety of somatic cells under controlled circumstances in vitro, it is very uncommon for them to transform into target cells after they have been transplanted. Additionally, in response to the hostile milieu, transplanted MSCs go through senescence or apoptosis [95]. Owing to the hostile microenvironment generated by injured organs or tissues, there are only a finite number of functioning stromal cells accessible for transplantation following MSC-based therapy and this presents a challenge for the treatment [96]. The acute inflammatory response that occurs in vivo serves as an efficient stimulant for the recruitment of progenitor cells. On the other hand, persistent inflammation poses a considerable barrier to the recruitment and survival of both naturally present progenitor cells and transplanted MSCs. As a result, paracrine and anti-inflammatory processes are the primary contributing factors in the repair of liver tissue injury and enhancing survival in animal models with liver damage [10].

**Fig. 3:**
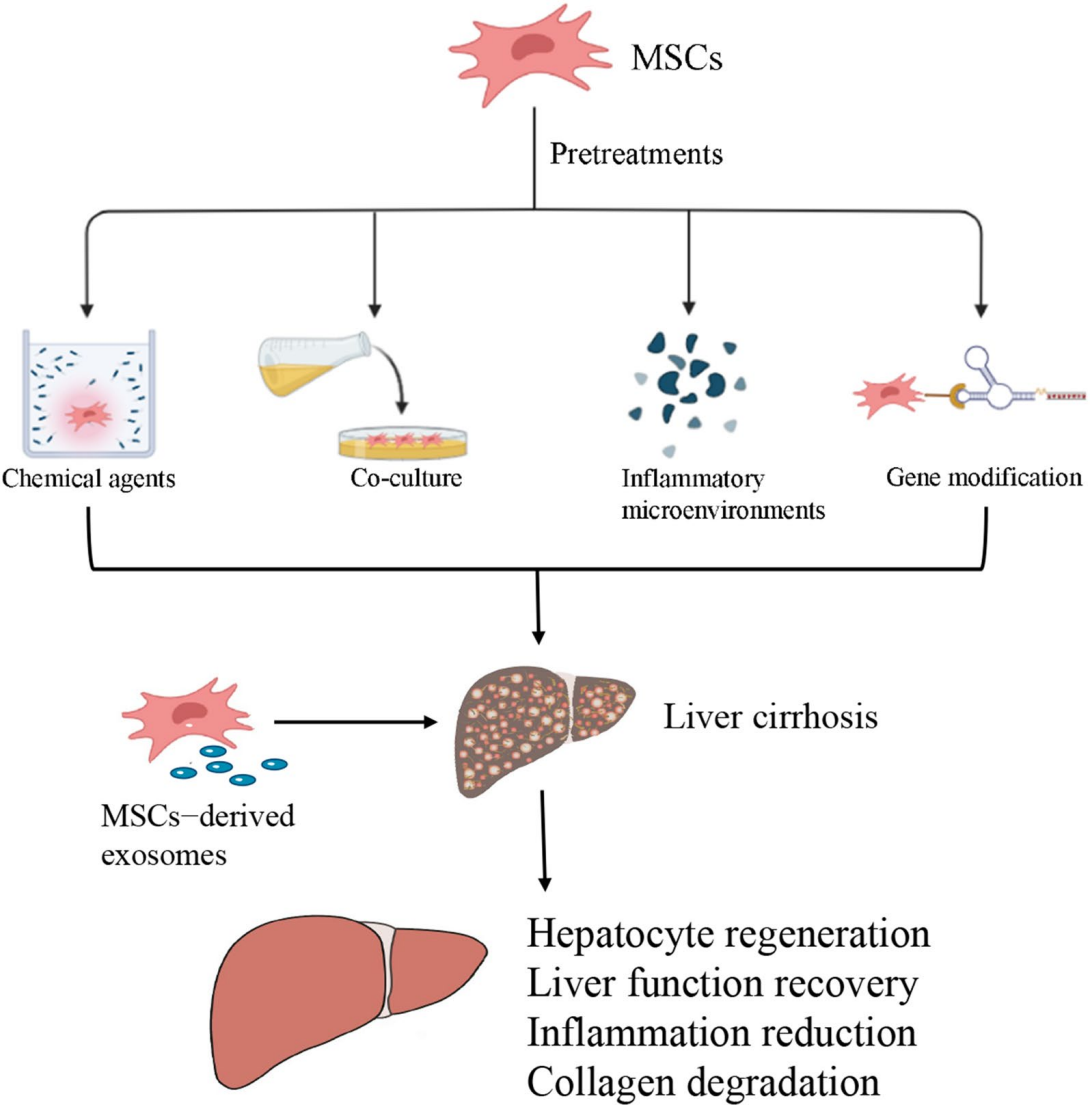
Pretreatments enhance the therapeutic effects of MSCs in liver cirrhosis

Studies have shown that gene modification, pharmacologic agents, hypoxia, and inflammation milieu may all be used to shield MSCs from the damage that is caused by a hostile milieu, hence enhancing the homing ability of MSCs, their rate of survival, their paracrine impacts in vivo and in vitro, and also the therapeutic effectiveness of these cells within the setting of liver cirrhosis [97]. In conventional cell culture, the oxygen level is typically approximately 21% O_2_; however, this oxygen level is not equivalent to levels present in the in vivo milieu. The normal concentration of oxygen in tissues ranges from 1 percent in bone marrow up to 12 percent in peripheral blood [98]. Hypoxic environments have been shown to drastically enhance the MSCs’ survival rate in the hostile conditions of injury sites upon transplantation. This has been demonstrated to positively affect MSC production of cytoprotective molecules, proliferation, and multipotency [99]. Mortezaee et al. [100] treated BM-MSCs with melatonin for 24 h before injection into the rat model of liver fibrosis and found that MSCs that had been pre-treated with melatonin exhibited a greater homing ability into the damaged liver regions. Additionally, there was a considerable improvement in the proportion of glycogen storage, and the accumulation of collagen and lipids in fibrotic liver tissue was greatly reduced. Watanabe et al. [88] displayed the differences and interactions between bone marrow-derived macrophages (id-BMMs) and MSCs in the mouse model of cirrhosis and found that combined treatment with MSCs and id-BMMs exhibited a synergistic effect with greater amelioration of liver cirrhosis and facilitation of hepatocyte regeneration, compared to the treatment with each cell alone. Ye et al. [101] demonstrated that in the CCl_4_ liver cirrhosis model, the therapeutic impact of BM-MSCs that had been transfected with hepatocyte nuclear factor 4 alpha (HNF-4α) was superior to that of BM-MSCs without treatment. The transplantation of HNF-4α-BM-MSCs alleviated liver damage as evidenced by an elevation in the levels of cytokeratin-18 (CK-18) and albumin, reduction in the expression levels of alanine transaminase (ALT), bilirubin total levels, and aspartate aminotransferase (AST), as well as the decrease in inflammation associated with the reduction in IL-6, IFN-γ, and TNF-α, and the suppression of Kupffer cells (Fig. 3).

## MSCs-derived exosomes for treating liver cirrhosis

Research shows that the EVs generated by MSCs, such as exosomes (40–100 nm in diameter) and microvesicles (MVs, 0.1–1 mm in diameter), could make a significant contribution to the therapeutic potential of MSCs by facilitating cell–cell interactions and delivering paracrine factors during angiogenesis, tissue repair, and immunomodulation [102, 103]. Exosomes are nanoscale EVs derived from multivesicular bodies (MVBs). Exosomes are secreted into the extracellular microenvironment as a result of the fusion of MVBs with the plasma membrane. These exosomes may either be taken up by target cells that are located in the milieu or transported to distant areas through biofluids [104]. Exosomes are known to contain a broad spectrum of cytoplasmic as well as membrane proteins encompassing ECM proteins, lipids, transcription factors, receptors, and nucleic acids (miRNA, mRNA, dsDNA, ssDNA, and mtDNA) [105]. Exosomes have been shown to perform an instrumental function in a wide range of cell–cell interaction pathways, which are connected with a diverse range of pathophysiologic activities [106].

Several different animal models of liver illnesses, such as drug-induced acute liver damage and liver fibrosis, have been observed to benefit from the injection of mesenchymal stromal cell-derived exosomes (MSCs-Ex) [107, 108]. Injection of MSCs-Ex into the livers of mice exposed to CCl_4_ resulted in a reduction in the severity of the fibrosis that had developed by inhibiting the production of collagen and TGF-β1 [109]. Jiang et al. [110] demonstrated that human umbilical cord MSCs-Ex reduced CCl_4_-mediated acute liver damage, as well as liver fibrosis, which was achieved by attenuating hepatocyte apoptosis and oxidative stress. Additionally, these MSCs-Ex enhanced the hepatoprotective and antioxidant properties of bifendate. MSCs-Ex also decreases the deposition of collagen and ameliorates CCl_4_-mediated liver fibrosis by suppressing EMT, alleviating liver inflammation, and eliminating hepatocyte apoptosis [107]. Moreover, MSCs are capable of releasing immunologically potent exosomes, which enables them to have immunoregulatory impacts on the differentiation, activation, and functionality of various subsets of lymphocytes [111]. Tamura et al. [112] revealed that MSCs-derived exosomes enhance the production of anti-inflammatory cytokines and the levels of T-regulatory cells in murine with concanavalin A-elicited liver damage, providing evidence for the immunosuppressive properties. The potential for MSCs-Ex to perform a function in the delivery of medications is another area in which they might be used in a therapeutical manner. Recent research has shown that MSCs are capable of packaging and delivering active agents via their exosomes. This opens the door for the use of MSCs in the research and development of novel pharmaceuticals that are more effective and have more homing potential [113].

Cell-free-based treatment methods eliminate the possible tumorigenesis, unnecessary differentiation, emboli formation, cell injection, and infection transmission that are associated with MSCs transplantation. Additionally, these treatment methods are safer, less expensive, and more successful [114]. The absence of fabrication methods that are both reproducible and effective continues to be a significant barrier, even though MSCs-Ex has significant promise in the treatment of liver illnesses. As a result, there is a need for the development of more effective techniques for the extraction, characterization, purification, and preservation of exosomes that are applicable in therapeutic settings. Meanwhile, to further understand the role that MSCs-Ex play in liver regeneration, more research is required. Research conducted on large animal models is required before the results can be implemented in clinical settings.

## Challenges and future directions

During the past two decades, numerous cell therapy technologies have emerged to treat liver cirrhosis. These techniques have resulted in the accumulation of knowledge regarding the enhancement of in vitro cell manipulation as well as the processes through which transplanted cells can counteract liver fibrosis and enhance liver repair. This information is now facilitating the development of novel techniques targeting the production of in vitro systems that potentially result in the creation of liver organoids or perhaps full bioengineered livers for transplant, with the help of established cutting-edge technologies such as bioreactors, microfluidics, and 3D (biology) printing [115]. The transplantation of MSCs has been evaluated in multiple clinical studies, and the findings have been encouraging and demonstrate MSCs transplantation as one of the potential alternative methods for treating patients who suffer from liver disorders. However, there have been reports of chromosomal abnormalities occurring in cultured cells, although research has shown the transplantation of MSCs to be safe in both preclinical settings and clinical trials [116]. Because of this, questions about the safety of therapies based on MSCs are still being discussed, particularly in the context of long-term follow-up. The key worry is undesired differentiation of the transplanted MSCs and their propensity to impair anti-tumor immune reaction and develop new blood arteries that could facilitate tumor development and spread [27]. Furthermore, the most effective method of administering MSCs is not yet uncovered, and their use in clinical trials has not yet been standardized to this day. When reviewing the findings from clinical studies, one of the practical issues that must be addressed is the optimal dosage, as well as the number of injections. In addition, there are not yet any advanced tools available for monitoring engrafted MSCs. In summary, the quality of the clinical investigations that have been published so far is insufficient to arrive at an outcome that can be considered definitive.

## Conclusions

Therapy based on mesenchymal stem cells (MSCs) is a potential approach among the several medical procedures now in use for the treatment of liver illnesses and could be employed accordingly to give the best match for therapy, in an attempt to prevent the frequently occurred fatal consequence of the necessity for liver transplantation. On the other hand, to optimize the therapeutic value of MSCs, mechanistic as well as clinical investigations should create closer cooperation between academic and industrial researchers.

## Data Availability

All data are included in this published article.

## Abbreviations

MSCs: Mesenchymal stem cells
ASH: Alcoholic steatohepatitis
NASH: Non-alcoholic steatohepatitis
ECM: Extracellular matrix
HRS: Hepatorenal syndrome
HLCs: Hepatocyte-like cells
MHC: Major histocompatibility
HSCs: Hepatic stellate cells
TGF-β: Transforming growth factor-β
PDGF: Platelet-derived growth factor
α-SMA: α-Smooth muscle actin
TIMP: Tissue inhibitor of matrix metalloproteinases
MMP: Matrix metalloproteinases
ISCT: International society for cell therapy
TNF-α: Tumor necrosis factor alpha
IL: Interleukin
IFN-γ: Interferon gamma
EVs: Extracellular vesicles
HGF: Hepatocyte growth factor
PD: Programmed death
PGE2: Prostaglandin E2
TLR: Toll-like receptor
DCs: Dendritic cells
NK: Natural killer
dsRNA: Double-stranded RNA
OSM: Oncostatin M
CCl_4_: Carbon tetrachloride
CM: Conditioned medium
HNF-4α: Hepatocyte nuclear factor 4 alpha
ALT: Alanine aminotransferase
AST: Aspartate aminotransferase
iNOS: Nitric oxide synthase
MVBs: Multivesicular bodies
EMT: Epithelial mesenchymal transition
